# Perspectives of mothers and fathers affected by addiction of an adolescent/adult child – results from a mixed-method study

**DOI:** 10.1186/s13690-025-01826-7

**Published:** 2026-01-10

**Authors:** Anja Bischof, Richard Velleman, Stefan Borgwardt, Hans-Juergen Rumpf, Gallus Bischof

**Affiliations:** 1 Department of Psychiatry and Psychotherapy, University of Lübeck, Lübeck, Germany; 2 Department of Psychology, University of Bath, Bath, United Kingdom; 3 Sangath Community Health, Raia, Goa India

**Keywords:** Affected family members, Substance use, Psychological stress, Strain, Social support, Coping, Barriers to treatment

## Abstract

**Background:**

Addiction has an impact not only on the individual but also on family members. Stress and strain are high especially in parents of individuals with an addiction. Aim of the study is to focus on the experiences of parents and to integrate their view in a model for a better understanding of psychosocial burdens of addiction-affected families.

**Methods:**

The multi-modal study included qualitative in-depth semi-structured interviews following a guideline based on the Stress-Strain-Information-Coping-Support Model and additional quantitative assessment. Participants were recruited via self-help groups for parents (nationwide and regional). Data of 21 mothers and 9 fathers were analysed in terms of gender differences.

**Results:**

While mothers were more affected by emotional and cognitive stress factors, fathers suffered more from violent conflicts. Mothers tended more towards stressful coping strategies such as taking responsibility and tabooing the addiction, whereas fathers were more able to distance themselves from the addiction problem. For mothers, feelings of guilt and shame were a major barrier to seeking help; for fathers, the lack of admission of their own helplessness played a significant role. Both groups expressed the wish for extended support, better access to the help system, intensified media awareness, and advertisement of easy-to-access emergency help.

**Conclusions:**

Feelings of guilt, shame, and fear of stigmatisation were identified as key barriers to seeking professional help. The Stress-Strain-Information-Coping-Support Model of how family members are affected by addiction was enlarged to include ‘barriers to’ and ‘needs for’ support.

**Supplementary Information:**

The online version contains supplementary material available at 10.1186/s13690-025-01826-7.

**Table:** 

Text box 1. Contributions to the literature
1. Affected family members of individuals with addiction problems, a significant group of the population, suffer from poorer health and consequently generate higher healthcare costs.
2. To date, there is little information available about parents who are confronted with their children's addiction problems, especially with regard to dealing with the issue in terms of the gender of the parent.
3. Our results contribute to a better knowledge about the needs of parents and the barriers to treatment and therefore to an improvement of targeted interventions.

## Background

The negative impact of addiction on the health of addiction-affected family members (AFMs) has been consistently demonstrated in various studies [1]. AFMs show increased rates of affective disorders, anxiety disorders, reduced health status, increased medical treatment costs, and productivity losses [2–5]. In addition, insurance data from the U.S. show that increased medical treatment costs of family members revert to the reference population when abstinence is achieved by the individual with an addiction. This indicates increased morbidity and treatment needs of AFMs as a direct result of the substance use disorder/substance misuse [6, 7]. Although it is not possible to estimate prevalence rates worldwide, since most previous studies use multipliers or other approximation parameters to estimate the prevalence, the public health impact of being an AFM is immense [8].

There is insufficient scientific evidence in relation to two specific dimensions. First, although a recent nationwide study estimated the proportion of parents among AFMs in Germany at 10.3% [5], much less is known about parents than about partners and spouses: a significant number of studies have focused mainly on physical harms to partners and spouses as a consequence from substance use [9, 10]. Second, although there is evidence that the burden and coping strategies of AFMs differ depending on gender [11, 12], there is insufficient scientific evidence on gender-specific differences between parents. The majority of studies on family members focus on female partners and mothers [13], and yet 43.1% of AFMs in a German representative study were male [5], suggesting a lack of information about male AFMs. As a result, it is impossible to make reliable statements about differences between mothers and fathers.

Parental roles are strongly influenced by gender roles which – depending on cultural prerequisites and influences – inform normative role obligations (e.g. [14]). Traditional role attributions in the parenting unit (the father as financial provider, the mother as the main care-giver of children) may also have led to a bias in research concerning family bonding and responsibilities in families [15, 16] such as that mothers have a closer relationship to their child. Previous research reports significant parental stress, especially for mothers, which results from societal expectations towards parents who are held responsible for the development of their child [15–17]. This can lead to increased and severe stress and burden, resulting in self-blame, shame, and self-stigmatisation among parents of an individual with addiction [18–22].

Parents suffer from intensified worries about their offspring’s substance use, fear for their child’s health and future, and increased helplessness in coping with the addiction problem [22–25]. Not wanting to acknowledge the problem in the beginning, coupled with little support from the social environment, and a lack of communication from therapeutic institutions when their child is in treatment, all lead to increased burden for parents [26]. The fear of stigmatisation and of being blamed can lead to isolation [27] and are considered barriers to seeking treatment [28, 29]. Furthermore, there are often significant communication problems between mothers and fathers about how the family might or should cope with substance use disorders (SUDs) in a child [30], which leads to additional family stress.

The present work intends to contribute to a broader understanding of both the psychosocial stress situation of parents of adult/adolescent children (i.e. index patient, IP) with an addiction, and of gender differences between mothers and fathers. Based on previous qualitative research, we were interested in assessing gender differences regarding stress factors, coping behaviour, psychological and somatic strain as well as barriers and attractors of help-seeking. In a second step, we aimed to integrate the results of the present study into Orford et al.‘s Stress-Strain-Information-Coping-Support Model (SSICSM; [3]). The model postulates that regular interaction with an individual suffering from addiction represents a stressful situation that leads to strain on the AFM; with this strain being influenced by the type of information, coping and support available or utilised. For a more detailed description see Velleman et al. [31]. In addition to the existing components of the model (stress, strain, information, coping, support), used as a guideline for the interviews and assessed with open questions, we wanted to explore in depth the parents‘ needs and barriers to seeking help. Although previous studies have demonstrated substantial strain in AFMs, only a minority of them seek help. Although professional help is available in many countries, so far studies focussing on AFMs have not yet addressed either the specific needs arising from that strain, or the barriers that might stop AFMs from seeking support. There are some overlaps between ‘specific needs’, and ‘barriers’ and the existing components of stress, strain, coping and support, but it seemed clear to us that these aspects are not adequately addressed by the existing model. We assume that these needs and barriers can contribute significantly to the model. The identification of support needs can make it possible to adapt care offers to the needs of parents in a more goal-oriented way and may contribute to an improvement in utilisation rates and treatment compliance of parents in the long term. The identification of barriers within the help system could be used to modify help offers in the sense of low-threshold offers and destigmatisation of the group of AFMs.

## Methods

The present study was part of the mixed-method study „Burden, Expectancies, Perspectives of Addicted individuals‘ Significant others“ (German: Belastungen und Perspektiven Angehöriger Suchtkranker: BEPAS), which recruited participants via cooperating self-help groups and via pro-active screening in general practices and general hospitals in Germany. The present analysis was restricted to AFMs recruited from self-help groups and consisted of 30 parents (21 mothers and 9 fathers, including two step-fathers).

### Recruitment via self-help groups for AFMs

Through self-help groups, people with certain relationship status and gender attributes can be recruited specifically. The cooperating self-help groups (SHGs) were not following a ‘Fellowship’ (as e.g. Al-Anon) model in terms of the support they offered. Facilitators of SHGs for affected family members were asked to approach group members to take part in the study. Those who agreed were put in touch with the research team. We aimed to recruit AFMs that had not attended a SHG for more than 12 months to avoid a possible bias caused by IPs with severe chronic dependence and therefore more strain in AFMs. A total of 66 eligible participants from SHGs were interviewed, including 30 parents. The cooperation with self-help groups allowed us to employ a theoretical sampling approach: the leaders of the self-help groups were asked to invite participants with special characteristics (e.g. specific addiction forms like gambling or specific groups like men or fathers). Therefore, we could include specifically a subgroup of fathers which previous research has shown to be difficult to reach.

### Procedure

A brief telephone interview was held with all potential AFMs prior to study participation to check inclusion criteria (age 18 to 64, addiction problem of the IP active within the last 12 months, at least 15 to 20 h of personal contact per week) and assess information on the relationship constellation and the addiction problem. The subsequent interviews (June – December 2016) took place at the AFMs’ convenience: in their homes, in the cooperating self-help facilities, or in the premises of the University Hospital in Lübeck. The interviews were always held with just the AFM, without the IP. The IP was not part of the study and only knew about it if the parents actively informed them. The study focused solely on the parents’ experiences. If there were more than one AFM, they were interviewed separately. The interviews were conducted by staff members (psychologists or a comparable degree) who are trained in qualitative interviews. All AFMs received information sheets with general information about the project and data protection. To increase the participation rate, all AFMs received an incentive of 50€.

At the beginning of the interview, the interviewer asked for sociodemographic data (sex, age, marital status, relationship to the IP) and general information about the addiction problem. The subsequent in-depth interviews were recorded using a recording device and summarised within 24 h. The interview took on average 93 min (range: 62 to 130 min, standard deviation: 16.8). Additionally, quantitative questionnaires were administered after the narrative interview. During the interview, an empathetic approach was central. The participants were encouraged to illustrate emerging topics with concrete examples whenever possible. All participants gave informed consent. The study was conducted according to the Declaration of Helsinki and was approved by the Ethical Board of the University of Lübeck.

### Guideline for the qualitative interview

The qualitative interview used an interview guideline, based on studies by Orford and colleagues (e.g. [32]) and the SSICSM. The overarching themes of the modified guideline were: the negative impact of substance use on the AFM, the AFM‘s coping attempts, and formal and informal resources. Additionally, open questions on support needs and barriers to seeking help were integrated into the guideline. The interview guideline can be found in Appendix 1.

### Assessment

The main focus of the study was on the qualitative interviews: the quantitative data was only descriptive and subordinate. The quantitative questionnaires were only administered after the qualitative interviews in order not to influence the narrative. The coding process of the qualitative interviews was carried out independently from the qualitative analysis. The assessment included sociodemographic data and two standardised questionnaires used in previous studies on AFMs [33]: the Short Questionnaire for Family Members – Affected by Addiction (SQFM-AA; [34–36]) and the Hopefulness-Hopelessness Scale (HOPE; [33]). Additionally, we used the Patient Health Questionnaire (PHQ-8; [37]), the Oslo-3-Items-Social-Support Scale (OSSS-3; [38]), and a brief questionnaire for the assessment of generalised self-efficacy (ASKU; [39]).

### Analysis

During the interviews, the interviewers took detailed accounts and wrote down verbatim quotes, alongside noting important keywords and recorded the interviews with a recording device. Both audio files and interviewer reports were analysed. Similar to the approach taken by the UK Alcohol, Drugs and the Family Research Group, these materials were used to create a usually two-page ‘interview report’, prepared within 24 h of the interview. As in the studies by Orford and colleagues [11], a complete transcription of the interviews was not carried out, as the informational added value of this additional effort was considered to be comparatively low. Instead, we focused on documenting specific examples and verbatim quotations in order to illustrate the stressful situation of relatives as vividly as possible. All quotes were anonymised before inclusion in this paper.

Based on the audio files and the reports of the interviewers, the analysis of the interviews was carried out in the form of an iterative group process between assessment and analysis according to Content Analysis [40] and Grounded Theory [41, 42]. While stress factors, coping, and support were assessed using open-ended questions based on previous studies focussing on the elements of the SSICSM, no prior knowledge was available for barriers and needs. These were therefore assessed using open-ended questions and added to the model using open coding. Our approach therefore included elements of both analysis approaches.

In the first stage of the study, interviews were openly coded by at least two raters and results were compared in meetings. Inconsistencies were resolved in group discussions until consensus was achieved. During team discussions, relevant topics were extracted. This resulted in a continuously growing list of topics and categories.

The final 179 items were structured in six main- and 24 sub-categories. The items for every interview were rated with 0 = criterion not met, 1 = criterion partially met, 2 = criterion fully met. All interviews were coded by at least two independent raters and discussed in case conferences. In terms of inter-rater reliability, Cohen’s Kappa coefficient was almost perfect (0.81–100) in 25 categories, substantial (0.61-0.80) in 63 categories, moderate (0.41-0.60) for 65 categories, and fair (< 0.40) for 22 categories.

Statistical descriptive analysis of the questionnaires was carried out with IBM SPSS Statistics 28. Group comparisons were calculated with Mann-Whitney-U-tests. A significance level of *p* < .05 was defined as statistically significant.

## Results

The mean age of the 21 mothers was 53 years (range 42–65), that of the 9 fathers 55 years (range 49–61). 67% of the mothers and 100% of the fathers lived in a partnership. Nine of the mothers (38.1%) versus two of the fathers (22.2%) lived with the index patient. Daily contact with the IP was reported by 42.9% of the mothers compared to 33.3% of the fathers. The most common reported addiction problem of the IPs was cannabis use (83.3%), followed by use of other illicit drugs (30.0%), alcohol (30.0%), medication (20.0%), and disordered gambling (6.7%). Multiple addiction problems were reported in 56.7% of cases. Because not all parents knew what the IP consumed, it is not possible to be precise over the range of either ‘other illicit drugs’ or ‘medication’ that parents reported. The 30 participants were fathers and mothers of 26 IPs. Of the IPs, two were female. The IPs’ age was not collected systematically, but in the qualitative interviews, the parents (not always exactly) mentioned the age of their IP. The interviewer reports reveal that four of the IPs were minors (< 18 years old), while the majority of IPs were between 19 and 29 years old (two 18 years, one 41 years).

Both mothers and fathers demonstrated in the interviews similar, and quite severe, levels of both stress and strain, although mothers reported greater levels than fathers on some of the quantitative measures (see Table 1). As one mother said about herself and her husband: “We can’t get any rest anymore. Neither of us can sleep. We both have blood pressure problems” (participant 1019, mother of a son with multiple drug use). These stress and strain issues included the impairment of family and professional life, the strain caused by the IP’s unreliability and emotional unpredictability, communication problems (both with the IP, and between the parents), and the overall experience of ‘stress’.

**Table 1:** Strain, coping and resources (quantitative data)

	Total (*n* = 30)	Males (*n* = 9)	Females (*n* = 21)	*p*	ES
Family Members Assessment (SQFM-AA)					
Stress:					
Worrying behaviour, M (SD)	4.73 (2.63)	4.22 (2.82)	4.95 (2.58)	0.477	0.13
Active disturbance, M (SD)	2.23 (2.31)	3.00 (2.60)	1.90 (2.17)	0.240	0.21
Strain:					
Psychological strain, M (SD)	7.03 (1.79)	5.89 (1.62)	7.52 (1.66)	**0.017**	0.43
Physical strain, M (SD)	5.40 (2.65)	4.11 (3.02)	5.95 (2.33)	0.109	0.29
Coping:					
Engaged-emotional, M (SD)	4.89 (2.53)	4.22 (2.99)	5.14 (2.33)	0.425	0.15
Engaged-assertive, M (SD)	4.80 (2.48)	4.11 (3.10)	5.10 (2.19)	0.523	0.12
Tolerant-accepting, M (SD)	2.33 (2.56)	2.11 (2.80)	2.43 (2.51)	0.578	0.10
Withdrawal-independent, M (SD)	4.93 (2.80)	5.00 (3.08)	4.90 (2.76)	0.909	0.02
Support:					
Helpful informal, M (SD)	5.77 (3.17)	4.78 (3.80)	6.19 (2.86)	0.345	0.17
Helpful formal, M (SD)	4.07 (3.17)	4.11 (3.37)	4.05 (3.17)	0.873	0.03
Unhelpful informal, M (SD)	1.23 (1.43)	0.33 (0.71)	1.62 (1.50)	**0.014**	0.45
Total Family Burden, M (SD)	26.60 (11.22)	23.56 (13.07)	27.90 (10.41)	0.277	0.20
Sum score PHQ-8, M (SD)	6.9 (4.8)	4.2 (3.0)	8.0 (5.1)	**0.032**	0.39
Subjective health status, M (SD)	2.77 (0.86)	2.22 (0.83)	3.00 (0.78)	**0.017**	0.44
Life satisfaction, M (SD)	6.77 (1.94)	7.56 (1.3)	6.43 (2.09)	0.301	0.19
Sum score social support (OSLO-scale), M (SD)	8.20 (1.54)	7.56 (1.33)	8.48 (1.57)	0.075	0.33
Sum score self-efficacy (ASKU-scale), M (SD)	3.64 (0.67)	3.89 (0.73)	3.54 (0.64)	0.268	0.20
Sum score HOPE-scale, M (SD)	31.63 (6.77)	31.33 (6.34)	31.76 (7.09)	0.821	0.04

t-test for mean values and Mann-Whitney-U-test for statistical significance

*M* mean value, *SD* standard deviation, *ES* standardized effect size *r*

*Significance level: p<0.05*

Mothers and fathers differed in how they experienced the situation. Mothers more often reported feeling burdened on a psychological and affective-cognitive level, talking more frequently in the interviews of their feelings of guilt, of their concerns over the IP’s health, and of conflicts of loyalty, helplessness and the pressure to keep the addiction a secret. A mother of a son with cannabis use problems and gambling disorder described her feelings as follows: “I am in constant fear. A constant fear that is in me, that something might happen. Right now. That he could use again, gamble again. This constant fear that I can’t handle it anymore. Also, why I didn’t realise it” (participant 1018).

In addition, mothers more often reported fear, sadness and depressive moods that were directly related to the IP’s addiction, as shown in this mother’s quote: “I myself had a burnout and had to be in a psychosomatic clinic for six weeks. That was the point at which nothing worked anymore, when I went there. I realised, that I was becoming more and more physically depleted, I was getting thinner and thinner and was also pretty exhausted. Really, really exhausted.” (participant 3008, mother of a son with cannabis use disorder).

Fathers on the other hand were more burdened by the aggressive behaviour of the IP in the form of insults, threats, and vandalism. A father of a son with cannabis use disorder and psychosis described the damage caused by his son as follows: “He then went on the rampage because he couldn’t handle his anger. In the end, when he moved out, I had to replace two room doors, including the frames. I had to replace a flat door (fire door) that had been kicked off its hinges. … He threw a dumbbell into a wall with a deep hole in it and on the other side of the wall were the bathroom tiles. The force was so strong that it destroyed bathroom tiles on the other side. They had to be replaced. He crushed the shower screen and smashed the mirror. Stabbed into the ceiling with the barbell so that there were real holes in the walls and ceiling. And all that in a 10-year-old flat” (participant 2001).

These qualitative results from the narrative interviews were mirrored by the quantitative results, as depicted in Table 1. For example, in the scales and sub-scales of the SQFM-AA, mothers showed significantly higher levels of psychological strain. Similarly, mothers showed significantly greater levels of depression on the PHQ-8. None of the fathers, but seven of the mothers scored a value in the PHQ-8 indicating an at least moderate depression (sum score > 9). Moreover, mothers assessed their general health status as significantly poorer (5-point Likert scale; higher scores indicate poorer health).

No significant differences were found between mothers and fathers concerning hopefulness/hopelessness, perceived social support (both reported poor to moderate support measured with the OSSS-3), and self-efficacy which was comparable with reference values from the general population [39]. t-test for mean values and Mann-Whitney-U-test for statistical significance. M = mean value, SD = standard deviation. ES = standardized effect size *r*.

While both mothers and fathers discussed within the interviews how difficult it was to cope with having an IP with such a problem, there were again differences between their coping methods. Fathers reported being more inclined to distance themselves from the addiction problem: they took less responsibility for the IP and the problems that arose in the context of the addiction. Mothers, on the other hand, found it more difficult to distance themselves from the IP and the addiction problem: they took on more responsibility and sacrificed themselves for the IP. This could result in feeling divided between different personalities, as a mother of a son with cannabis use disorder reported: “You feel like you’re living two lives. You have to think for yourself about what you’re living, what needs to be done and taken care of during the day. And I had to think for my son, or had the feeling I had to think for him, about what he still had to do in his day, what he had to do and what he didn’t have to do. That was difficult and not at all feasible” (participant 3008). Another mother complained about the lack of action of her cannabis, cocaine, and amphetamines using son: “He shows virtually no initiative. You have to give him everything, you have to constantly take him by the hand, you have to push him, you have to guide him and if you don’t do it, then it won’t work” (participant 1010). In addition, they reported more often that they had hidden or concealed problems.

In terms of dealing with the addiction and creating or agreeing rules about the IP’s behaviour, fathers tended to agree on fewer rules and conditions, and then found it comparatively easier to implement these agreements consistently, as this father of a son with cannabis use disorder explained: “The attitude towards him has also changed, of course. In that way: I don’t care what you do. It’s your life. I’m not going to ruin my life because of you. If he thinks he has to live like this, he has to ruin it, then he has to ruin it. But I’m not going to let him get to me and ruin me” (participant 2007). Mothers reported more frequently that they changed their way of dealing with the addiction over time and developed strategies for self-care at a comparatively later stage.

Although both parents discussed needing support, there were differences in how they achieved this. Mothers described wanting to gain support from within the family. In contrast, fathers were more likely to seek support or distraction externally, for example within their work environment or by leaving the house and gaining strength and distracting themselves through exercise. This meant that often mothers perceived that they were getting less support than they wished from their partners, whereas fathers did not report this. While fathers saw their partners mostly as a backup, mothers rarely did: they often wished for more support from their partners and wished that they might more often handle the IP’s addiction as a couple. This could be a burden for mothers, as the following participant explained: “The problem for us as parents was often that we were not even in agreement. It would actually have been important for us as parents to agree on how to deal with the addiction and that one parent is not doing it one way and the other parent does something different” (participant 3007, mother of a son with cannabis use disorder). These disagreements often led to arguments, as another mother of a cannabis using son confirmed: “Especially in the beginning, I often disagreed with my husband and we argued, actually only about this topic’ (participant 2006).

Although fathers were more likely to gain support and relief from outside (exercise, work) it was mothers who were more likely to report having sought professional help, and mothers who expressed significantly more needs and who provided ideas for improving the addiction help system. Especially pronounced was the mothers‘ wish to get more concrete information both on how to deal with the addiction and via instructions for acute emergencies and problematic situations. Although fathers reported less use of professional help themselves, they were still supportive of the idea of providing more preventative help: both fathers and mothers expressed the wish for more information and counselling to be already available in schools to prevent the development of an addiction disorder, as well as more awareness in the public and in the media.

Mothers and fathers also differed in their view of the barriers blocking parents from accessing help. Mothers reported that the fear of stigmatisation was a significant barrier for the utilisation of professional help, as the following participant describes: “I think it’s probably hushed up by many. I don’t think I’m the only one who feels ashamed, where everything has to be kept up on the outside, but it doesn’t matter what it looks like on the inside” (participant 1019, mother of a son with multiple drug use). For fathers, on the other hand, the lack of admission of their own helplessness was more of an issue, as can be seen by the expressed despair of a father of a son with alcohol and cannabis use disorder: “I actually want to be a good father, but in the current situation I don’t know what that is. What is a good father in a situation like this?” (participant 1007, father of a son with alcohol and cannabis use disorder).

Mothers and fathers were equally dissatisfied with the undifferentiated, partly stigmatising portrayal of addiction in society and in the media: “What other people think about the parents of kids with an addiction? Losers!” (participant 3006, father of a son with cannabis use disorder). They expressed criticism of the lack of presence/visibility and networking of the addiction help system and saw that that a major problem was the lack of accessibility of support facilities, and the difficult access or long waiting times for further (therapeutic) support services, for the IP with these problems.

**Fig. 1:**
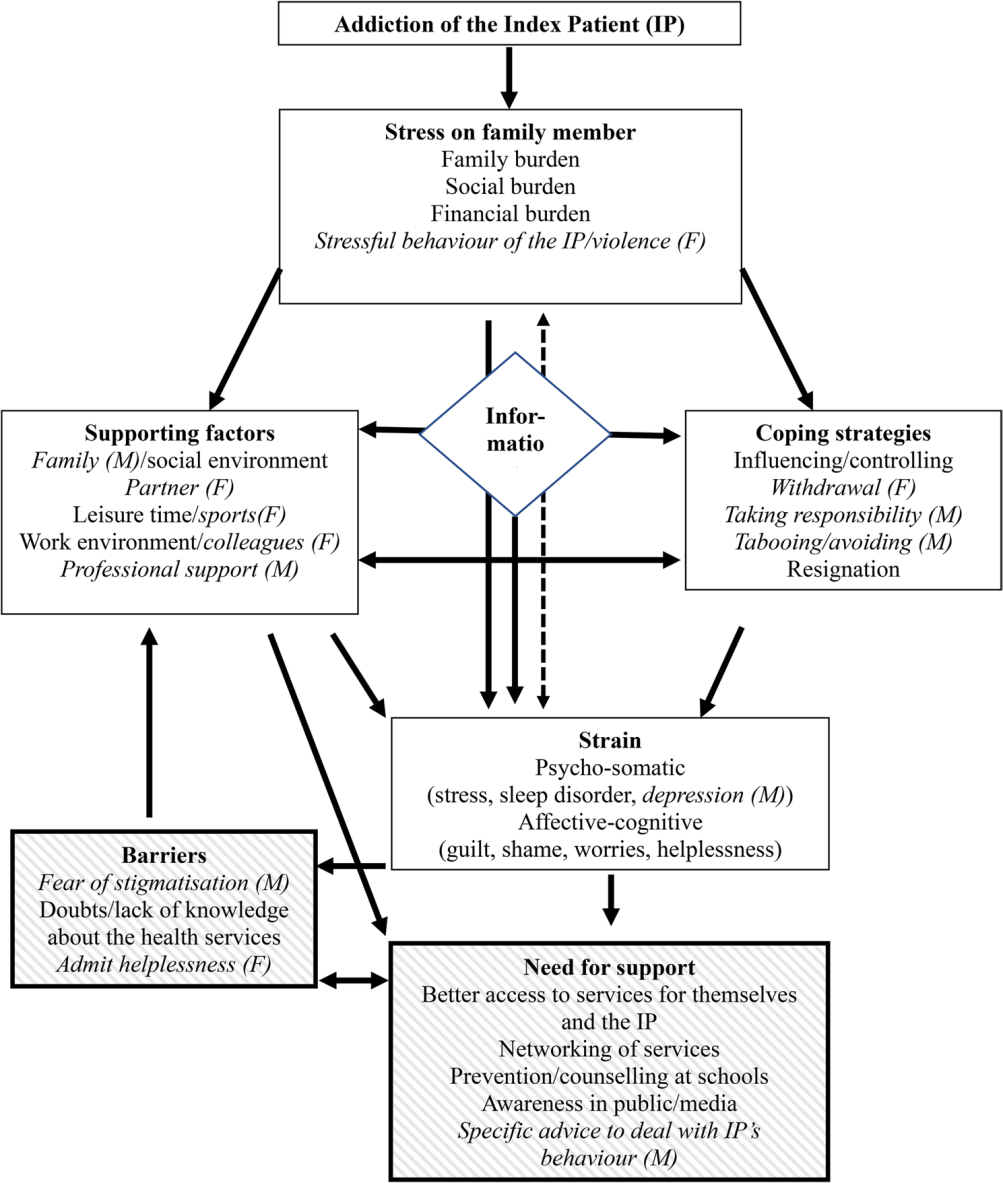
Extension of the Stress-Strain-Information-Coping-Support-Model (Orford et al. 2013). Gender differences are in italics and marked with M for mothers and F for fathers. The extensions of the model are marked with cross-hatching

The results of this study were used to further develop the Stress-Strain-Information-Coping-Support-Model (SSICSM; Fig. 1). Additionally to the core elements of the model, our study was able to show the importance of assessing support needs of parents and barriers to treatment. These can be integrated into the model.

Parents have specific needs that are currently not yet covered with the existing model, which, when taken into account, could lead to an enhancement of seeking support and an improvement in coping with the addiction. At the same time, parents expressed barriers to treatment like shame and lack of knowledge about the various support options. In order to inform stakeholders and health policies and to improve the support system, an expansion of the model seems necessary. This is emphasized by a lack of research on the support needs of family members of individuals with an addiction [43].

Gender differences were found in the following aspects of the model: Concerning stress, fathers emphasized that they were strongly burdened by stressful or violent behaviour of the IP. Concerning support, fathers were more likely to find support and relief from their partner, their colleagues, and in doing sports, mothers were more likely to find support in family and in professional services. Although the coping strategies influencing/controlling and resignation showed no differences, fathers were more likely to use withdrawing while mothers more often either took responsibility or tried to avoid the topic of addiction. In terms of strain, mothers more often showed depressive symptoms. While a major barrier to treatment for mothers was the fear of stigmatisation, to fathers the barrier was over admitting their helplessness. In terms of needs, mothers more often expressed their wish to get specific advice on how to deal with the addiction problem.

## Discussion

The wide range of stresses and strains, and of coping strategies, shows the complexity of the psychosocial stress situation of parents of individuals with an addiction. The impairment in family life due to the necessity of intensive care for the IP was perceived similarly by mothers and fathers. However, differences were found in many stress factors, adverse consequences, and coping strategies.

The gender-specific differences in psychological strain (e.g. fear, depression) were confirmed by the quantitative data. Fathers felt more able to give responsibility back to the IP and to distance themselves emotionally from them. Mothers, on the other hand, were found to be more willing to sacrifice themselves for the IP, a mechanism that often occurred after a history of failed coping attempts. This may have led to a neglect of their own needs and is probably grounded in a (self-) expectation of how the mother-role should be fulfilled [15, 44]. Overall, mothers in similar stressful situations became more involved in the IP’s problems caused by the addiction compared to fathers. They were more inclined to use coping mechanisms that led to stress-related psychological strain, such as taking on responsibility for the IP or hiding the addiction problem. This is underlined by a questionnaire-based study from Sweden with 687 parents (14% fathers) that reported a perceived greater responsibility of mothers resulting in a stronger burden in the female subsample [24].

Another difference between mothers and fathers related to the support each wished for and received from their spouse. Whereas being in a partnership was often seen as a helpful resource for fathers, mothers sometimes perceived their partner as an additional burden. This aspect may also have contributed to increased stress for mothers. Although these results relate to how the different genders within a parental couple see themselves, they also mirror the results from studies which focus on female experiences with their relative’s substance abuse or gambling behaviour. In general, studies on AFMs haven shown females to be more burdened by a relative’s addiction problem [45]. This may relate to the fact that, in many cultures, mothers are the main care-givers responsible for the IP’s well-being, and feeling responsible for, and self-sacrificing towards, the IP is common [16, 17, 46, 47]. This can be traced back to a historically and socially conditioned understanding of gender role attributions in the family [15]. Additionally, living together with the IP – which in our study occurred almost twice as much with mothers than fathers (40% vs. 22%) – can increase the burden on AFMs [36].

In our study, for both parents, fear of stigmatisation played an important role, both in their experience of stress and as a barrier to seeking help. This is in line with data from a Canadian study where parents reported to have withdrawn from the extended family and from friends [26], as well as in a Swedish study where feelings of shame and guilt and the fear to be stigmatised were barriers to the help system [28]. Related to a societal concept of family, stigmatisation and negative self-evaluation is not uncommon, as a narrative review by Smith and Estefan elaborated [15]. Self-blame for being a “bad parent” and feeling guilty for the offspring’s addiction problem is common among parents [22, 23]. For the mothers in our study that reported to have considered seeking help, fear of stigmatisation (especially the fear of negative reactions or the fear of being held responsible for the addiction) appeared as a central barrier to seeking help; and also as a major precursor to tabooing, avoidant behaviour and accordingly to utilising coping strategies that are associated with higher stress, such as hiding the addiction problem from the social environment. This is in line with previous research on the topic of coping with an addiction problem for parents [22, 48]. Fathers on the other side less often reported considering seeking help and thus less often reported conscious barriers for help-seeking.

The need for professional support, which was more frequently verbalised by mothers, was expressed by many in the desire for concrete behavioural instructions over how best to deal with the IP. This could be related to the strongly perceived helplessness in handling the addiction problem and a more pronounced sense of guilt. Nevertheless, there were very few gender-specific differences over the support needs mentioned: mothers and fathers were largely unanimous in their wish for extended support for both the IPs and themselves, improved access to the help system, intensive public awareness campaigns by the help system, a more differentiated presentation of addiction in the media, as well as prevention and counselling offers at schools.

The results are helpful to augment the original SSICSM. The model has been developing over the years, from an early “Stress-Coping-Model” [49] via a “Stress-Coping-Health-Model” [50] and a “Stress-Strain-Coping-Support-Model” [51] to a “Stress-Strain-Information-Coping-Support-Model” [3]. The current study contributes to a next stage of the model development, adding a B for barriers to seeking or accessing help and an N for needs for support, resulting in a “Stress-Strain-Information-Coping-Barriers-Needs-Support-Model” (SSICBNSM). These additions can help to raise awareness of the special needs and barriers of AFMs and to develop ways of providing help that are appropriate to the difficult situation in which AFMs find themselves.

### Limitations

Despite an intended over-recruitment of male AFMs, a gender balance was not achieved. Additionally, we cannot rule out the possibility that the participating fathers are not representative of the entire group of fathers, as participation in a support group already indicates a willingness to reflect on and discuss one’s own problems. In addition, the sample was drawn from an unrepresentative, limited population, as all were recruited through cooperating self-help groups. Nevertheless, the analysis of the interviews took place in parallel with the survey and there were major thematic overlaps in the realised interviews, so that we assume that we have meaningful findings for the target group addressed, but of course these cannot be generalised for parents who do not attend a SHG or have other characteristics such as migration background. Furthermore, no statements for AFMs who have little or no contact to the help system can be made. Parents visiting a SHG are likely to be parents of IPs with an already chronic addiction disorder, so it can be assumed that they are more burdened than parents of IPs in an early stage of substance abuse. Our sample was rather homogenous and located in Germany. Therefore, to examine cultural differences was not an aim of the study. Even though traditional gender roles are no longer as pronounced in Germany, they still play a significant role in sociocultural terms (women as caregivers, men as providers).

When interviews were scheduled, parents were asked to describe the type and severity of the addiction problem of the IP. An independent clinical assessment of the disorder could not be conducted. Therefore, the study relies only on subjective perceptions of the parents. However, the aim of the study was to describe the parents’ experiences and their perceptions of the seriousness of their child’s addiction, as opposed to relying on the clinical manifestations of the IP’s disorder.

The gender distribution of the IPs was also unbalanced. Only two parents reported that they had a daughter with an addiction; all other IPs (*n* = 24) were male, which does not correspond to the actual gender ratio of individuals with an addiction [52]. In addition, the presence of the addictive disorder of the IP was not specified on the basis of the IP’s assessment of their own use, nor on any external diagnosis but was the subjective assessment of the parents. Finally, the small sample size does not allow for generalisability and must be taken into account when interpreting the quantitative results. Additionally, gender differences in strain, coping, and health in the quantitative questionnaires might be overlooked because the sample sizes were too small to show them. The sample size was not predetermined to detect statistical significances. At the same time, lack of statistical differences therefore does not indicate equality of the groups. Differences in PHQ-8 might reflect gender differences not specific for AFMs. Due to the design of our study, we did not include matched controls unexposed to addiction in the family. Finally, experiences of AFMs and parents in particular could have been influenced by other traits that were not examined in this study like personality, family constellations, social background, and resilience.

## Conclusions

One aim of the BEPAS study was to use the above-mentioned perceptions of barriers and needs to generate hypotheses for how the support system can be improved for AFMs. Parents – as a subgroup of AFMs – suffer from similar stress and strain as other family members [53]. However, feeling responsible for their son or daughter is likely to increase the burden, although direct comparisons between partners and parents are scarce [54]. Additionally, other groups of family members other than parents or partners are seldomly subject to research [12]. Nevertheless, research on specific stress factors for the different AFM groups is lacking. Our subgroup-specific results of the present study may provide initial indications of the essential support needs of parents. From the multitude of needs verbalised in the interviews, the following essential aspects could be identified: the desire for concrete behavioural guidelines, better accessibility and simplified access to the help system, a change in public awareness of addiction, and intensive prevention and education in schools. Feelings of guilt, shame, and fear of stigmatisation were identified as key barriers for mothers to seeking professional help; for fathers, the lack of admission of their own helplessness played a significant role.

In order to meet the parents’ desire for individual behavioural advice in concrete problem situations, it might be helpful to set up specialised telephone hotlines for AFMs that could be contacted in acute emergency situations. Improved online services that provide information and counselling, such as those already available for those affected by addiction themselves that also aim to address stigma [55], would also be helpful. At present, there is a sparsity of online help specialised in dealing with the topic of AFMs in general and certainly not for parents of individuals with an addiction, who are often in desparate need for advise, information, and help to get their offspring into treatment [48]. Additionally, some form of couples’ work involving mediation or counselling could be helpful, since the desire to handle the IP’s addiction problem as a couple was expressed by many mothers within the present sample. For many parents, it seems to be important to have tailored counselling that individually addresses their personal needs. Community Reinforcement and Family Training (CRAFT; [56]) and the „5-Step Method“ [57] are two evidence-based and increasingly popular approaches that address this concern. Previous studies have shown that these approaches effectively address the individual needs of family members of IPs, even if the IP is not ready for treatment [58–62]. The desire for an increased public awareness of addiction and help services for AFMs should be met by providing easily accessible information, as well as work on altering public perceptions and media representations of addiction.

This latter would also work to counteract a major barrier to accessing help: the fear of stigmatisation. Many interviews revealed that parents were ashamed and afraid of being blamed for the addiction; and feelings of shame and guilt can contribute to parents wanting to cope with the problem alone and refraining from seeking professional help. The removal of the taboo surrounding addiction as a result of an increased social debate could lead to relief and a greater willingness to access treatment among parents affected by a loved one’s addiction. Such work on altering public perceptions and representations could take the form of positive role models in the media and publicity campaigns aimed at removing blame from family members and also pointing out the comparatively good prognosis of addictive problems. At the same time, training specialists and policy makers in strategies to overcome stigmatisation of substance use problems as well as of families living with addiction, is needed [29].

In addition, barriers to treatment based on shame could be countered by promoting low-threshold clinical early intervention services. For young people with addiction problems and their parents, access could be made easier by early help offers to prevent the intensification and chronification of addiction problems, such as a close networking of youth facilities and addiction counselling services, including social workers and street workers. Furthermore, it would be useful to install counselling services directly in schools. Close cooperation between teachers, parents and addiction counselling services seems appropriate and should be expanded through further training of teachers. Although most of the IPs were not minors at the time of the interviews, the majority of the IPs had a history of substance use dating back to school days. This is why the parents emphasized the need for services in schools and more prevention – not for themselves, but for other affected parents.

Many parents reported multiple substance use of the IP and additional comorbidities (especially other mental health concerns) that made the parents unsure which „port of call“ would be the right one for the IP, since many addiction counselling services only specialise in certain forms of addiction, and many other counselling services focus on one mental health problem (e.g. depression) and exclude people with substance problems. Counselling centres could counteract this uncertainty by improving the flow of information and training of professionals, especially regarding comorbid addiction-related disorders. Finally, interventions that are informed by our conclusions of course need to be evaluated in further studies.

## Supplementary Information

Supplementary Material 1.

## Data Availability

The datasets used and/or analysed during the current study are available from the corresponding author on reasonable request.

## Abbreviations

AFM: Addiction-affected family member
ASKU: Allgemeine Selbstwirksamkeit Kurz-Skala (assessment of generalised self-efficacy)
BEPAS: Burden, Expectancies, Perspectives of Addicted individuals‘ Significant others
CRAFT: Community Reinforcement and Family Training
HOPE: Hopefulness-Hopelessness Scale
IP: Index patient
OSSS-3: 3-item Oslo Social Support Scale
PHQ-8: Patient Health Questionnaire
SHG: Self-help group
SQFM-AA: Short Questionnaire for Family Members – Affected by Addiction
SSICSM: Stress-Strain-Information-Coping-Support Model
SUD: Substance use disorder
