# Supplementary material for: Perspectives of mothers and fathers affected by addiction of an adolescent/adult child – results from a mixed-method study
Source: Arch Public Health. 2026 Jan 10;84:21. doi: 10.1186/s13690-025-01826-7 (PMC12849084; doi:10.1186/s13690-025-01826-7)
Supplement: Supplementary file 1 — Supplementary Material 1. [file 13690_2025_1826_MOESM1_ESM.docx]

**Interview guideline for the qualitative interview BEPAS**

1. **HISTORY AND CHARACTERISTICS OF THE ADDICTION PROBLEM**

**Short description of the addictive behaviour of the index patient (IP) and it’s development.**

**Specific informations on the addictive behaviour (type, duration, consumption place, quantity, sources).**

**If addictive behaviour is currently weak: last typical consumption phase.**

- 1. Age of IP / relation to the family member affected by addiction (FMA)
  2. Type of addiction - substance?
  3. Extent of addictive behaviour, quantity, frequency, patterns?
  4. Places where substances are consumed? At home? Alone? In front of the family?
  5. Financial burden of the addictive behaviour?
  6. In case of illegal drugs: Sources / Procuring the substances?
  7. Development of the addictive behaviour? Abstinence? Relapses?
  8. Utilisation of professional help (of the IP)?
  9. Description of the FMA’s consumption or gambling patterns?

1. **CONSEQUENCES FOR FAMILY MEMBERS**

**General description of the effects of the addictive behaviour.**

**What are the worries/fears? How are burdens experienced?**

**In the end, it should be clear why consumption is a problem and what the FMA is concerned about.**

**Important: Elaborate / Verbatim quotes and examples of concrete incidents / behaviour**

- 1. What is living with the IP like? Problems? Worries?
  2. Impact on the relationship to the IP (arguments, tensions, violence)?
  3. Impact on family interactions?
  4. Impact on social life?
  5. Impact on family finances / standard of living / theft?
  6. Embarassement / feelings of shame?
  7. Impact on the FMA’s well-being (physically & mentally)? Sleep/anxiety/stress?
  8. Impact on the well-being of other family members?
  9. Impact on the FMA’s own consumption/gambling behaviour?
  10. **Additional burden besides the addiction problem (e.g. due to work)?**
  11. **Acute/medium-term/long-term effects?**

1. **COPING WITH THE ADDICTIVE BEHAVIOUR**

**Get a complete overview of the FMA’s coping attempts and the IP’s responses.**

**Which coping strategies have been experienced as particularly helpful?**

**Assess modifications of coping strategies over the time.**

**Important: Ask for examples and elaborate.**

- 1. Description of coping attempts
  2. Certain attitude patterns? Being strict, paying more attention to themselves?
  3. Behavioural patterns? Setting rules? Attempts to control? Cancel contact?
  4. Suitable / unsuitable coping strategies?
  5. Desirable coping strategies, that are difficult / have not been possible until now?
  6. Changes in the interaction with the IP? Which? Why?

1. **RESOURCES / EXPERIENCED SUPPORT**

**Assess resources / support that has been experienced as helpful. Get an overview of the FMA’s social network / environment.**

- 1. Helpful resources? What / who gives strength?
  2. Support by members of the household? Handling the problem together? Collaboration?
  3. Support by others outside of the family? Who? Collaboration?
  4. Influence of local aspects? E.g. neighborhood?

1. **NEEDS / BARRIERS**

**Expected support that has not been received yet?**

**Needs / wishes towards the help system?**

- 1. Utilisation of professional help (self-help groups for FMAs, counselling…)? What has been helpful, what hasn’t been? **When / how / by whom was the help been utilised?**
  2. **Were there barriers to utilisation? What did hinder the FMA?**
  3. How do others think about FMAs? Image? What role does this play in terms of utilisation?
  4. Need for support? What is requested? By whom? Ideas for improvement? Access ways?
